# HBV-Related Hepatocellular Carcinoma Susceptibility Gene *KIF1B* Is Not Associated with Development of Chronic Hepatitis B

**DOI:** 10.1371/journal.pone.0028839

**Published:** 2012-02-21

**Authors:** Rong Zhong, Yao Tian, Li Liu, Qian Qiu, Ying Wang, Rui Rui, Bei-Fang Yang, Sheng-Yu Duan, Jun-Xin Shi, Xiao-Ping Miao, Li Wang, Hui Li

**Affiliations:** 1 Education Key Laboratory of Environment and Health, Department of Epidemiology and Biostatistics and Ministry, School of Public Health, Tongji Medical College, Huazhong University of Science and Technology, Wuhan, Hubei, China; 2 Department of Epidemiology and Biostatistics, Institute of Basic Medical Sciences, Chinese Academy of Medical Sciences; School of Basic Medicine, Peking Union Medical College, Beijing, China; 3 Department of Epidemiology and Biostatistics, School of Public Health, Guangdong Pharmaceutical University, Guangzhou, Guangdong, China; 4 Department of Virology, Wuhan Centers for Disease Prevention and Control, Wuhan, Hubei, China; National Cancer Center, Japan

## Abstract

**Background:**

A recent genome-wide association study has identified a new susceptibility locus, kinesin family member 1B gene (*KIF1B*), strongly associated with progression from chronic hepatitis B (CHB) to hepatitis B virus-related hepatocellular carcinoma (HCC) in Chinese population, this study was carried out to explore the role of the genetic variants in *KIF1B* in the development of chronic hepatitis B.

**Methodology/Principal Findings:**

Three *KIF1B* polymorphisms (rs8019, rs17401924, and rs17401966) were selected and genotyped in 473 CHB patients and 580 controls with no history of CHB. Odds ratios (ORs) and 95% confidence intervals (CIs) were calculated by logistic regression model. None of these three SNPs showed association with CHBs after adjusting for age and gender. Equivalence-based method analysis confirmed the absence of association. In the further haplotype analysis, three common haplotypes were observed in this study population, but no significant effect was also found for haplotypes in the progression to CHB.

**Conclusions/Significance:**

This study showed the new locus identified for HCC, *KIF1B*, was not associated with progression to CHB, implying distinct genetic susceptibility factor contributes to the progression from hepatitis B virus infection to HCC. Nevertheless, further comprehensive analyses are warranted to dissect the mechanism.

## Introduction

Hepatitis B virus (HBV) infection is one of most challenging global public health problems, with more than 2 billion people infected [1], [2]. Epidemiological studies have shown a regional diversity of prevalence, with low rate (0.1–2.0%) in the USA and western Europe and high rate (8.0–20.0%) in southeast Asia and sub-Saharan regions [1]. While in China, a national survey in 2006 reported a 7.18% rate of HBsAg in the general population aged 1–59 [3], [4], [5]. HBV infection leads to a wide spectrum of outcomes. It has been estimated that 90–95% of individuals can successfully clarify HBV, and only 5–10% would develop chronic hepatitis B (CHB) [6]. Among those CHB individuals, 20–30% would develop liver cirrhosis and 5% further progress to hepatocellular carcinoma (HCC) [7]. HCC is the third most common cause of cancer-related death, and more than 80% of HCC cases can be attributable to chronic infection with HBV in hyper-endemic regions, suggesting CHB was a major risk factor for development of HCC [8]. The enormous variation in clinical outcome of HBV infection highlights the importance of identification of mechanism underlying the progression of HBV exposure to CHB for prevention against HBV-induced fatal liver disease. Although the environmental factors such as alcohol abuse, infection age, and co-infection with other hepatitis virus unveiled as risk factors of HBV-induced liver disease, genetic factors may also influence clinical progression after HBV exposure, which is indicated by familial studies [9]. In fact, multiple candidate genes, such as *IFNG*, *TNF*, *VDR*, and *HLA* loci, have been extensively investigated in the progression to CHB, but results were inclusive [10]–[13]. A recent genome-wide association study (GWAS) by Kamatani et al. in Japanese population has suggested two SNPs of rs3077 and rs9277535 in HLA-DP region strongly associated with risk of persistent infection of HBV [14]. Immediately, we successfully validated these SNPs in two Chinese independently population [15]. However, these two variants could account for only a small portion of CHB, and additional variants remains to be identified.

Interestingly, a GWAS conducted in Chinese population by Zhang H et al. raised a new susceptibility locus (rs17401966) in kinesin family member 1B gene (*KIF1B)* at chromosome 1p36.22, which was reported to be associated with HBV-related HCC in six independent populations [16]. However, no comprehensive analysis has been performed to explore this genetic variant on the progression of CHB. Given the established relationship between HBV infection and HCC, a hypothesis was therefore raised naturally that this new identified loci was also involved on the progression of CHB. To test this hypothesis, a case-control study containing 473 CHB patients and 580 controls matched in age and gender with no history of CHB, was conducted to explore the associations between the progression to CHB and three candidate SNPs in *KIF1B*, one SNP rs17401966 from GWAS study [16], and two SNPs of rs8019 and rs17401924 from functional prediction analysis.

## Materials and Methods

### Study subjects

This study consisted of 473 CHB cases and 580 controls. All subjects were unrelated Han Chinese from Beijing city and its surrounding region. CHB patients were recruited from two infectious diseases hospital, Beijing Ditan Hospital and Beijing You'an Hospital between November 2001 and August 2004. The diagnosis of CHB was conducted based on HBsAg seropositive, anti-HBs seronegative, and continuously abnormal alanine aminotransferase (ALT) and asparate aminotransferase (AST) for more than six months. Controls matched in age and gender were selected from a physical examination in Peking Union Medical College Hospital in 2004, who had no medical history of chronic hepatitis B and HBV vaccination. Subjects who were positive for HBsAg were excluded from the controls. At recruitment, written informed consent was obtained from each subject and personal information from each participant regarding demographic characteristics such as gender, age, and history of HBV vaccination were collected by questionnaire. This study was approved by the institutional review boards of the Chinese Academy of Medical Sciences Cancer Institute and Tongji Medical College of Huazhong University of Science and Technology.

### Serological testing

Enzyme-linked immunoadsorbent assay (ELISA) was used to detect the serum HBsAg, anti-HBs and anti-HBc (IMX; Abbott Diagnostics, North Chicago, IL, USA).

### Identification of Candidate SNPs

In addition to the SNP of rs17401966 identified by the GWAS in Chinese population, two other candidate SNPs in the intron of *KIF1B* gene were identified by bioinformatics analysis. The SNP of rs8019, which may located in the binding site of microRNA, was selected using an integrated bioinformatics tool “SNP Info”(http://manticore.niehs.nih.gov/snpfunc.htm) [17]. And rs17401924 was predicted to influence the gene splicing, which was analyzed by “ESE finder” (http://rulai.cshl.edu/cgi-bin/tools/ESE3/esefinder.cgi?process=home) [18].

### Polymorphism genotyping

Genomic DNA was extracted from peripheral blood lymphocytes. All subjects were genotyped using TaqMan real-time polymerase chain reaction (Applied Biosystems, Foster City, CA) without knowledge of subjects' infection status. The program was heating to 95°C for 10 minutes followed by 40 cycles of 95°C for 15 seconds and 60°C for 1 minute. The ABI Prism 7900HT Sequence Detection System was used to read the reacted plates and analyze the endpoint fluorescence. To ensure the accuracy of genotyping, a 15% random sample was tested twice by different investigators. The results were concordant for all of the duplicate sets.

### Statistical analysis and haplotype construction

The differences in distribution of genotype between CHB cases and controls were tested by χ^2^ test. Logistic regression was used to evaluate the associations between *KIF1B* genotypes or haplotypes and the outcome of HBV infection. Odds ratios (ORs) and their 95% confidence intervals (CIs) were calculated after adjustment for age and sex where appropriate. Nonsuperiority test was used to confirm the absence of association between all SNPs detected and progression to CHB [19]. All statistical analyses were conducted by Statistical Analysis System Software (version 9.2; SAS Institute, Cary NC). Linkage disequilibrium (LD) of the three SNPs was estimated using Haploview [20]. Haplotypes were reconstructed and Haplotype frequencies were analyzed using Phase 2.1 [21].

## Results

### Subjects characteristics

A total of 473 chronic HB patients (377 males and 96 females) and 580 controls (462 males and 118 females) were enrolled in this study, with mean age of 34.13±13.56 years. Both age and gender distribution was comparable between CHB and controls. The baseline characteristic of CHB patients and controls were shown in Table 1.

**Table 1:** Distributions of select characteristics among CHB patients and controls.

Variable	Controls (%)	CHB patients (%)	*P* value
Total	580	473	
Age at enrollment, years	34.43±13.75	33.75±13.34	0.415
Gender			
Male	462 (79.7)	377 (79.7)	0.984
Female	118 (20.3)	96(20.3)	

### KIF1B SNPs genotypes and risk of CHB

The genotype distributions of *KIF1B* candidate SNPs are shown in Table 2. The minor allelic frequencies (MAF) for the rs17401966, rs8019 and rs17401924 variants were 0.26, 0.38, and 0.27 in CHB patients and 0.28, 0.37, and 0.28 in controls, respectively. No significant differences were found in the genotype frequencies between CHB patients and controls. Also, no significant association was observed between the rs17401966 AG (OR = 1.01; 95% CI, 0.79–1.31) or GG genotype (OR = 0.70; 95% CI, 0.43–1.14) and progression to CHB after adjusting for age and gender using logistic regression analysis (table 2). Similarly, the other two SNPs, rs8019 and rs17401924, also showed no significant association with progression to CHB (table 2).

**Table 2:** *KIF1B* genotype frequencies and ORs for the association with CHB in Chinese population.

Genotype	control (*n* = 580)	CHB (*n* = 473)	OR (95% CI)^a^	*P* value
	N (%)	N (%)		
rs8019A→C				
AA	232 (40.0)	177 (37.4)	Reference	
AC	265 (45.7)	230 (48.6)	1.14 (0.87–1.48)	0.339
CC	83 (14.3)	66 (14.0)	1.04 (0.72–1.52)	0.835
AC+CC	348 (60.0)	296 (62.6)	1.11 (0.87–1.43)	0.396
rs17401966A→G				
AA	305 (52.6)	254 (53.7)	Reference	
AG	225 (38.8)	190 (40.2)	1.01 (0.79–1.31)	0.915
GG	50 (8.6)	29 (6.1)	0.70 (0.43–1.14)	0.152
AG+GG	275 (47.4)	219 (46.3)	0.96 (0.75–1.22)	0.723
rs17401924A→G				
AA	307 (52.9)	249 (52.6)	Reference	
AG	222 (38.3)	192 (40.6)	1.07 (0.83–1.38)	0.625
GG	51 (8.8)	32 (6.8)	0.78 (0.48–1.24)	0.290
AG+GG	273 (47.1)	224 (47.4)	1.01 (0.79–1.29)	0.928

a Data were calculated by unconditional logistic regression, adjusted for sex and age.

### Haplotypes and risk of CHB

We further performed haplotypes analysis of these three SNPs (in the order of rs8019, rs17401924, and rs17401966, data is showed in Table 3). LD analysis showed that these three SNPs were in strong linkage disequilibrium, with a D′ of 0.972 (r^2^ = 0.595) for rs8019 and rs17401924, 0.921 (r^2^ = 0.524) for rs8019 and rs17401966, and 0.931 (r^2^ = 0.853) for rs17401924 and rs17401966 in our study population. Eight haplotypes were observed in the present study, of which, only three haplotypes AAA, CGG and CAA were the most prevalent haplotypes both in CHB patients and controls owing to strong LD in these three SNPs. Haplotype analysis found that individuals carrying the CAA or CGG haplotype showed no increased risk of progression to CHB with reference of the AAA haplotype. Meanwhile other five haplotypes composing these three SNPs with frequencies smaller than 5% would not be evaluated in the haplotype association study.

**Table 3:** Distribution of haplotype frequencies among CHB patients and Controls and their association with CHB in Chinese population.

Haplotype^b^	Controls*n* = 580	CHB patients*n* = 473	OR (95% CI)^a^	*P* value
	No. of chromosomes (%)	No. of chromosomes (%)		
AAA	720 (62.1)	565 (59.7)	Reference	
CAA	107 (9.2)	106 (11.2)	1.26 (0.94–1.69)	0.115
CGG	312 (26.9)	227 (24.0)	0.93 (0.76–1.14)	0.466

a Data were calculated by unconditional logistic regression, adjusted for sex and age.

b The haplotypes were in the order of rs8019, rs17401924, and rs17401966, and haplotypes with frequencies <5% were not shown in the table.

### Equivalence-based method to confirm the absence of association between KIF1b SNPs and risk of CHB

Nonsuperiority test was used to confirm the absence of association of rs8019*C, rs17401966*G, and rs17401924*G with the progression of CHB. The null hypothesis is that the frequency of rs8019*C, rs17401966*G, and rs17401924*G in CHB patients is greater by Δ compared to the frequency in controls. The specified amount Δ was set by 5% because the lowest difference of rs17401966*G between CHB and HCC patients in five independently populations in the GWAS data were 7.2%. So, a 5% excess in cases can be regard as a stringent margin.

The corresponding nonsuperiority *P*-values for rs8019, rs17401966 and rs17401924 was 0.002, 0.05 and 0.017, respectively, which support the absence of association between these three SNPs and development of CHB.

## Discussion

In the study, we conducted a case-control study to firstly investigate whether a new genetic susceptibility locus associated with HBV-related HCC identified by GWAS, *KIF1B*, was involved in development of CHB. However, none of the three candidate SNPs (rs8019, rs17401924, and rs17401966) in *KIF1B* was significantly associated with the progression to CHB, and equivalence-based test confirmed the absence of association.

Since CHB has been verified to be closely associated with HCC, identification of the genetic predictors of CHB development is very important for fighting against HBV-related HCC. Although researchers have paid much attention to identify genetic susceptibility loci to CHB during past decades, the specific genetic alteration underlying progression to CHB was far from clear. Recently, a new susceptibility locus, *KIF1B* rs17401966 at 1p36.22 region, was identified to be involved in the progression from CHB to HCC by a GWAS study in Chinese population [16], but the effect of this SNP was unclear in the progression from HBV exposure to CHB. In this study, we investigated this SNP and other two predicted functional SNPs in *KIFIB* in 473 CHB cases and 580 controls with no medical history of CHB, and no significantly associations were observed between these three SNPs and the progression to CHB after adjusting age and gender. The results indicate that the genetic variants in *KIF1B* may only play role in the progression from CHB to HCC, but not in the progression to CHB. Similarly result was found by a recent GWAS in Japanese that a identified genetic locus for hepatitis C virus-related HCC was not associated with chronic hepatitis C susceptibility [22], implying that distinct genetic mechanism may contribute to corresponding step of HCV-induced HCC development . The genome-wide analyses on loss of heterozygosity (LOH) in HCC by Li et al. further support our current result, which suggested the LOH of 1p36.21–36.32 region was significant related to HCC development, but not associated with the progression to CHB [23]. Noteworthy, the new identified *KIF1B* locus for HCC is located at 1p36 region, which encodes two spliced isoforms of kinesin protein (KIF1Bα and KIF1Bβ) involved in the transport of organelles and vesicles [24]. Of these two isoforms, KIF1Bβ has been elucidated to act as a tumor suppressor in multiple cancers by acting a variety of inhibitors of cell proliferation and/or activators of apoptotic cell death [25], [26]. Moreover, because a non-significant difference test cannot be interpreted as acceptance of the null hypothesis, equivalence-based method that provide the probability of observing a lack of association by chance was conducted to avoid the false-negative results in this study. *P*-values of nonsuperiority test for rs8019, rs17401966 and rs17401924 is 0.002, 0.050 and 0.017, respectively, and the nonexistence of association between *KIF1B* and CHB progression is confirmed. Taken together, our current result, plus these published evidence, outlined a hypothetical theory that variable genetic susceptibility mechanisms underlie different procedural steps from HBV infection to hepatocarcinogenesis, and strongly indicated that the genetic variants in *KIF1B* significantly contributed to the progression of CHB to HCC, but not the CHB susceptibility.

In conclusion, this current study highlights the specificity of the *KIF1B* genetic variants for the progression from CHB to HCC, and the genetic susceptibility mechanism underlying the progression to CHB emergently needs further comprehensive analyses to elucidate.
